# Supplementary material for: Electron Bernstein waves driven by electron crescents near the electron diffusion region
Source: Nat Commun. 2020 Jan 9;11:141. doi: 10.1038/s41467-019-13920-w (PMC6952373; doi:10.1038/s41467-019-13920-w)
Supplement: Supplementary file 2 — Description of Additional Supplementary Files [file 41467_2019_13920_MOESM2_ESM.pdf]

Electron Bernstein Waves driven by Electron Crescents near the Electron Diffusion Region

W. Y. Li[1,2,3], D. B. Graham[2], Yu. V. Khotyaintsev[2], A. Vaivads[4], M. André[2], K. Min[5], K. Liu[6], B. B. Tang[1], C. Wang[1], K. Fujimoto[7], C. Norgren[8], S. Toledo-Redondo[9,10], P.-A. Lindqvist[4], R. E. Ergun[11], R. B. Torbert[12], A. C. Rager[13,14], J. C. Dorelli[14], D. J. Gershman[14,15], B. L. Giles[14], B. Lavraud[9], F. Plaschke[16], W. Magnes[16], O. Le Contel[17], C. T. Russell[18], & J. L. Burch[19]

[1]*State Key Laboratory of Space Weather, National Space Science Center, Chinese Academy of Sciences, Beijing 100190, China*

[2]*Swedish Institute of Space Physics, Uppsala SE-75121, Sweden*

[3]*State Key Laboratory of Lunar and Planetary Sciences, Macau University of Science and Technology, Macau, China*

[4]*Division of Space and Plasma Physics, School of Electrical Engineering and Computer Science, KTH Royal Institute of Technology, Stockholm SE-11428, Sweden*

[5]*Department of Astronomy and Space Science, Chungnam National University, Daejeon 34134, Republic of Korea*

[6]*Department of Earth and Space Sciences, Southern University of Science and Technology, Shenzhen 518055, China*

[7]*School of Space and Environment, Beihang University, Beijing 100191, China*

[8]*Department of Physics and Technology, University of Bergen, Bergen N-5020, Norway*

[9]*Institut de Recherche en Astrophysique et Planétologie, Université de Toulouse, CNRS, UPS, CNES, Toulouse 31028, France*

[10]*Department of Electromagnetism and Electronics, University of Murcia, Murcia 30003, Spain*

[11]*Laboratory of Atmospheric and Space Physics, University of Colorado, Boulder, Colorado 80303, USA*

[12]*Space Science Center, University of New Hampshire, Durham, New Hampshire 03824, USA*

[13]*Catholic University of America, Washington DC 20064, USA*

[14]*NASA Goddard Space Flight Center, Greenbelt, Maryland 20771, USA*

[15]*Department of Astronomy, University of Maryland, College Park, Maryland 20742, USA*

[16]*Space Research Institute, Austrian Academy of Sciences, Graz 8042, Austria*

[17]*Laboratoire de Physique des Plasmas, CNRS/Ecole Polytechnique/Sorbonne Université/Univ. Paris Sud/Observatoire de Paris, Paris 75252, France*

[18]*Department of Earth and Space Sciences, University of California, Los Angeles, California 90095, USA*

[19]*Southwest Research Institute, San Antonio, Texas 78238, USA.*

Supplementary data 1 legend

Parameters of the electron model distribution functions Columns from left to right: Number density ($N_{\rm r}$, in cm^{-3}), parallel and perpendicular thermal speeds ($\delta v_{\|}$ and $\delta v_{\perp}$, in km s^{-1}), the parallel speed ($v_{\|,\rm r}$, in km s^{-1}), and the perpendicular ring speed ($v_{\perp,\rm r}$, in km s^{-1}).
